# The quality of medical death certification of cause of death in hospitals in rural Bangladesh: impact of introducing the International Form of Medical Certificate of Cause of Death

**DOI:** 10.1186/s12913-017-2628-y

**Published:** 2017-10-02

**Authors:** Riley H. Hazard, Hafizur Rahman Chowdhury, Tim Adair, Adnan Ansar, A. M. Quaiyum Rahman, Saidul Alam, Nurul Alam, Rasika Rampatige, Peter Kim Streatfield, Ian Douglas Riley, Alan D. Lopez

**Affiliations:** 1 School of Population and Global Health, University of Melbourne, Level 5, Building 379, 207 Bouverie St, Carlton, VIC 3010 Australia; 2 School of Population and Global Health, University of Melbourne, Level 5, Building 379, 207 Bouverie St, Carlton, VIC 3010 Australia; 3 School of Population and Global Health, University of Melbourne, Level 5, Building 379, 207 Bouverie St, Carlton, VIC 3010 Australia; 4 Maternal and Child health Division, icddr,b, Mohakhali, Dhaka, 1212 Bangladesh; 5 Maternal and Child Health Division, icddr,b, Mohakhali, Dhaka, 1212 Bangladesh; 6 Health Systems and Population Studies Division, icddr,b, Mohakhali, Dhaka, 1212 Bangladesh; 7 Health Systems and Population Studies Division, icddr,b, Mohakhali, Dhaka, 1212 Bangladesh; 8 School of Population and Global Health, University of Melbourne, Level 5, Building 379, 207 Bouverie St, Carlton, VIC 3010 Australia; 9 Health Systems and Population Studies Division, icddr,b, Mohakhali, Dhaka, 1212 Bangladesh; 10 School of Population and Global Health, University of Melbourne, Level 5, Building 379, 207 Bouverie St, Carlton, VIC 3010 Australia; 11 University of Melbourne Laureate Professor, School of Population and Global Health, University of Melbourne, Level 5, Building 379, 207 Bouverie St, Carlton, VIC 3010 Australia

**Keywords:** Bangladesh, Death certificate, Cause of death, Medical certificate, Medical data audit, Vital registration system, Health policy

## Abstract

**Background:**

Accurate and timely data on cause of death are critically important for guiding health programs and policies. Deaths certified by doctors are implicitly considered to be reliable and accurate, yet the quality of information provided in the international Medical Certificate of Cause of Death (MCCD) usually varies according to the personnel involved in certification, the diagnostic capacity of the hospital, and the category of hospitals. There are no published studies that have analysed how certifying doctors in Bangladesh adhere to international rules when completing the MCCD or have assessed the quality of clinical record keeping.

**Methods:**

The study took place between January 2011 and April 2014 in the Chandpur and Comilla districts of Bangladesh. We introduced the international MCCD to all study hospitals. Trained project physicians assigned an underlying cause of death, assessed the quality of the death certificate, and reported the degree of certainty of the medical records provided for a given cause. We examined the frequency of common errors in completing the MCCD, the leading causes of in-hospital deaths, and the degree of certainty in the cause of death data.

**Results:**

The study included 4914 death certificates. 72.9% of medical records were of too poor quality to assign a cause of death, with little difference by age, hospital, and cause of death. 95.6% of death certificates did not indicate the time interval between onset and death, 31.6% required a change in sequence, 13.9% required to include a new diagnosis, 50.7% used abbreviations, 41.5% used multiple causes per line, and 33.2% used an ill-defined condition as the underlying cause of death. 99.1% of death certificates had at least one error. The leading cause of death among adults was stroke (15.8%), among children was pneumonia (31.7%), and among neonates was birth asphyxia (52.8%).

**Conclusion:**

Physicians in Bangladeshi hospitals had difficulties in completing the MCCD correctly. Physicians routinely made errors in death certification practices and medical record quality was poor. There is an urgent need to improve death certification practices and the quality of hospital data in Bangladesh if these data are to be useful for policy.

**Electronic supplementary material:**

The online version of this article (10.1186/s12913-017-2628-y) contains supplementary material, which is available to authorized users.

## Background

Detailed and accurate hospital death certificates are a key component of a strong vital registration (VR) system [1]. VR systems inform health policy and allow health decision makers to direct resources towards locally-specific health problems [1]. Even though physician-certified death certificates serve as the gold standard in determining causes of death (COD), hospital death certificates have been shown to be of poor quality in a range of countries [1–4]. Incorrect or incomplete death certificates can misdirect efforts to tackle time-sensitive health issues and lead to erroneous conclusions from health data.

The quality of death certificates is influenced by a number of factors, including medical education, physician knowledge, and hospital resources [5]. While diagnostic capabilities of health facilities may vary, it is important that medical death certificates are completed to a minimum standard. As such, the World Health Organization (WHO) has released guidelines for the international form of the Medical Certificate of Cause of Death (MCCD or the “death certificate”) [6]. The death certificate has two parts: Part 1 is used for diseases or conditions that lead directly to death and Part 2 is for other significant conditions. The first line of part 1 is the immediate cause of death, which is required, and the lowest line is the underlying cause of death. A column for both parts is used to approximate the time interval between onset of a condition and death.

The common errors in completing death certificates in accordance with the WHO death certificate guidelines are shown in Table 1 [6]. First, there should be only one cause recorded per line in a death certificate. More than one cause per line can make it difficult to establish the sequence of events. Second, there should not be an incorrect or clinically improbable sequence of events leading to death. The underlying cause of death (UCOD), the disease or injury that initiated the sequence of events that led directly to death, is the basis for the compilation of mortality statistics. However physicians often report the direct COD instead of the UCOD. Third, death certificates should have an appropriate time interval between the onset of the condition and the date of death. The time interval should be entered for all conditions reported on the death certificate. Fourth, doctors are encouraged not to use abbreviations when certifying deaths. Abbreviations can have different meanings in different settings, so they can be easily misinterpreted. Fifth, death certificates need to be written with clear handwriting so that coders can assign the appropriate code from the 10th revision of the *International Statistical Classification of Diseases and Related Health Problems* (ICD-10). Sixth, physicians need to use consecutive lines in Part 1 when filling out the death certificate. The underlying cause should be the lowest line and the coders should be able to follow the sequence of events. Seventh, ill-defined or vague conditions should not be entered as the underlying cause because they provide little information to guide public health programs to design interventions. Common ill-defined conditions are “organ failure” and “septicaemia.” Symptoms and signs as well as the mode of dying should also be avoided since they are of no public health value. Lastly, injuries and poisonings should have clear external causes and indicate the intention (suicide/homicide or accident); neoplasms should include specification of the primary site, whether malignant or benign, primary or secondary, and histologic type.

**Table 1:** Common death certificate errors

Type of Error	
Multiple causes per line	
Incorrect/clinically improbable sequence of events leading to death	
Interval between onset and death not shown	
Abbreviations used in certifying death	
Illegible hand writing	
Leaving blanks between lines in part 1	
Ill-defined condition entered as underlying cause of death	
Intentions of injuries and poisonings not included	
Details of neoplasms not included	

Current death certificates in Bangladesh typically only have basic demographic information with a few lines dedicated to attributing cause of death. The current format does not comply with WHO guidelines and complicates efforts to improve the policy value of cause of death data. Previous research has shown that programs that train and refresh physicians in completing death certificates can improve the quality of medical records and death certificates [7–12].

In this study, we introduced the international MCCD criteria into hospitals in two districts in south-eastern Bangladesh. We maintained the structure of the Bangladesh death certificates but inserted the relevant criteria from the international death certificate. We trained hospitals physicians in completing the new death certificates. We also assigned project study physicians to rewrite a new death certificate and determine an underlying cause from medical record review and assess the quality of death certificates. This analysis assessed the quality of medical records, assessed the quality of death certification, and identified the likely leading causes of death in hospitals in rural Bangladesh.

## Methods

### Overview

This study was conducted as part of a National Health and Medical Research Council (NHMRC) multi-country research project. Initially, the study investigator discussed project objectives with senior hospital staff members. The death certificate used by public hospitals was not structured like the international death certificate, so the core components/parts of the international MCCD were incorporated, without affecting any information available on the present death certificate. Accordingly, a death certificate was prepared and supplied in every unit of the hospitals where death certificates were issued. Several workshops were organized to train 250 doctors how to complete the death certificates before starting the project activities. Through didactic and interactive lecture, the workshops oriented doctors and nurses about the principles of death certification and the requirements of good clinical record keeping for quality data. Trainees were provided with practice clinical cases to assign a COD. Periodic refresher courses with doctors and interns were also conducted by study physicians to improve certification practices. A booklet on cause of death certification was distributed among all doctors, and a laminated guideline was displayed in each doctor’s room [13]. The project staff compiled completed death certificates and photocopied the clinical records of the deceased including available laboratory investigations for subsequent analysis.

Two study physicians were briefed and trained about the different sections of the medical data extraction forms and gold standard criteria for diagnoses of main causes of death among neonates, children, and adults. They were trained how to review the MCCD and clinical records to complete the Medical Data and Audit Form (MDAF). They were also trained on the ICD-10 manual.

### Inclusion criteria

The study took place between January 2011 and April 2014 in the Chandpur and Comilla districts of south-eastern Bangladesh. The study initially included all 8 public hospitals (one secondary level Chandpur District hospital and 7 primary level Upazila Health Complexes) and 6 private hospitals (including the International Centre for Diarrhoeal Disease Research (icddr,b) hospital) operating in the district headquarters of Chandpur district. In 2011, only 404 deaths were identified in all of these hospitals which provided about 500 beds for a population of approximately 2.3 million. The study was thus expanded to the Comilla Medical College Hospital (tertiary level) which has 500 beds, a daily overnight hospital stay of 750, and helps serve a District population of 5.3 million.

All deaths in the study period that occurred in the selected hospitals and for which death certificates and medical records were available were included. Medical records and death certificates were photocopied for study purposes. Deaths were classified as neonates (first 27 days of life), children (28 days – 12 years) and adults (12 years and older).

### Death certification

Bangladesh routinely reports hospital deaths from a single line death certificate and uses these reports to describe hospital mortality nationally. We introduced the international death certificate, which has three sections:

1. Part 1 – including diseases or conditions directly leading to death and antecedent causes including underlying cause of death
2. Part II – Other significant conditions (contributing causes)
3. A column to record the appropriate interval between onset and death

The doctors were instructed to assign their diagnosis according to international rules which included 1) when there was only one cause identified, they would put them in the first line of part 1 of the death certificate 2) when two or more causes were identified, they would assess the causal relationship between them, and they would write the causally related causes in part 1 with the underlying cause on the lowest line. They would write the cause which contributed but was not causally related to main causes into part II of the death certificate as the contributory cause.

The study physician reviewed the hospital death certificates and clinical records to rewrite a new death certificate and allocate an underlying cause of death to the case for the purposes of this study. While doing this, the study physician was instructed to note whether they assigned a new underlying cause or just made a change in the order of diagnoses on the hospital death certificate.

### Medical data and audit form (MDAF)

The study developed a Medical Data and Audit Form (MDAF) that included 1) basic information of the hospital and the deceased 2) the same cause of death information from death certificate (verbatim) 3) cause of death from medical record review by the study physician 4) quality of clinical records – do the clinical data justify the clinical diagnosis; was it necessary to change the clinical diagnosis or sequences in the diagnosis to allocate the UCOD? The quality of medical record review-based diagnosis was assessed according to gold standard diagnosis criteria used by the Gates 13 Grand Challenges in Health (GC13) study. This study used the gold standard criteria developed by the Population Health Metrics Research Consortium (PHMRC) [14]. These gold standard criteria were developed by a committee of physicians and underwent multiple cycles of group review. The gold standard criteria classified the deaths into six levels based on the degree to which the information from medical records provided certainty to classify the death for a given cause: level 1) Gold standard 1 (GS1): highest level of certainty- diagnosis of a particular condition with the highest level of certainty possible for that condition, consisting of either an appropriate laboratory test or x-ray/imaging with positive findings and/or medically observed and documented appropriate illness sign(s) to a pre-determined standard. 2) GS2A: high level of certainty – diagnosis of a particular condition with a high level of certainty, consisting of appropriate laboratory/x-ray with positive findings and /or medically observed and documented appropriate illness or signs to a pre-determined standard. 3) GS2B: high level of certainty – presumed initial diagnosis of a particular condition with high certainty; this category was kept reserved for cancer and HIV patients on long-term treatment where initial data had been lost. 4) GS3: Reasonable level of certainty – medical diagnoses not supported by appropriate level of lab investigations but which meet established clinical criteria. 5) GS4: Unsupported – medical diagnoses not supported by the adequate observed and documented clinical evidence/criteria. 6) Other – ranking does not have an appropriate GS category.

### Analysis.

To identify the likely leading causes of death in Bangladeshi hospitals we categorized causes of death from study death certificates. Included death certificates had a valid age, sex, rank of quality, and study ICD-10 code. Age was categorised as neonate, child, and adult categories. The number of times the study physicians had to change the underlying cause of death or the order of diagnoses on the hospital death certificate was tabulated and stratified by age, hospital, and for the top 5 most frequent gold standard causes of death for adults, children, and neonates. The gold standard underlying COD was given as an ICD-10 code by the study physicians. The top 15, 10, and 3 most frequent ICD-10 codes for adult, children, and neonates, respectively, were given a text COD corresponding with the ICD code. All other ICD codes were given a text COD corresponding to the ICD chapter name (see Additional file 1). The frequency of GS levels was stratified by the same categories.

The frequency of the top 20 causes of death for adults, top 10 causes for children, and top 5 causes for neonates were tabulated. Gold standard COD given as an ICD-10 code was translated to text using the same mapping methodology, but with causes of death outside the top 20, 10, and 5 most frequent causes of death for adults, children, and neonates collapsed into the “other” category.

Common hospital death certificate errors were tabulated for each death certificate: multiple causes per line, change in diagnosis necessary, change in sequence necessary, interval between onset and death not shown, abbreviations used in certifying death, leaving blanks between lines in part 1, ill-defined conditions entered as underlying cause of death, and ambiguous poisoning deaths. Since hospital physicians tended to use “with”,“&”, “and”, “as a result of”, and “due to” delimiters when listing multiple causes of death on the same line, these delimiters were used to identify death certificates that included more than one cause of death on the same line in part 1. Death certificates were determined to have an interval between onset and death if every contributing cause of death had a corresponding interval. The presence of abbreviations on each line of each death certificate were tabulated by creating a subset that contained two or three letter words or known four letter abbreviations (“COPD”, “VLBW”, etc.). Common English words (“to”, “and”, etc.) were then removed. Death certificates that had a blank between lines in part 1 were tabulated. The first line of death certificates that contained the ill-defined keywords “failure,” “septicaemia,” “shock,” “cardio,” “brain,” and “old” were manually examined. Lines that did not include any other specific information were deemed ill-defined UCOD. Poisonings that did not indicate the type of poisoning or whether the death was accidental or suicidal were tabulated. The frequency of death certificates with any of the above errors or without an immediate cause of death (entry in the first line) was calculated. The frequency of any error except those that did not include a time interval was also calculated, as was the frequency of common abbreviations by age group.

## Results

The study included 4914 death certificates (3 cases omitted due to invalid age, sex, or medical record rank of quality): 2936 (59.7%) were adults, 463 (9.4%) were children, and 1515 (30.8%) were neonates. 20.5% occurred in the Chandpur District Hospital, 71.2% occurred in Comilla Medical College Hospital, 2.9% occurred in private clinics and hospitals, and 5.5% occurred in Upazila Health Complex (Table 2). 27.1% of medical records were considered to be of high quality (GS1, GS2A, and GS2B) and 72.9% were low of quality (GS3 and GS4).

**Table 2:** Medical record quality by age and location (*n* = 4914 deaths)

Category	Rank	Quality (%)^a^		Total Deaths (%)^b^
		High	Low	
Age	Adult	38.5	61.5	59.7
	Child	21.6	78.4	9.4
	Neonate	6.7	93.3	30.8
Location	Chandpur District Hospital	15.9	84.1	20.5
	Comilla Medical College Hospital	31.1	68.9	71.2
	Private Clinic/Hospital	31.2	68.8	2.9
	Upazila Health Complex	13.8	86.2	5.5

High Quality: GS1 (highest level of certainty), GS2A (high level of certainty), GS2B (high level of certainty)

Low Quality: GS3 (reasonable level of certainty), GS4 (unsupported)

^a^rows sum to 100%

^b^column sums to 100% within each category

### Quality of medical records

Only 6.7% of neonate medical records were of high quality, compared to 38.5% of adults and 21.6% of children (Table 2). Quality of medical records was highest for adult stroke (57.9%) and child gastroenteritis and colitis (48.6%) (Table 3). Quality of neonate and child medical records by cause of death was lower compared to that of adults.

**Table 3:** Medical record quality age and cause of death

Age	Rank	Quality (%)^a^		
		High	Low	Number of deaths
Adult	Acute myocardial infarction	24.5	75.5	424
	COPD	20.9	79.1	153
	Other cardiovascular diseases	28.2	71.8	174
	Other digestive diseases	32.5	67.5	154
	Stroke	57.9	42.1	463
Child	Encephalitis	0.0	100	34
	Gastroenteritis and colitis	48.6	51.4	35
	Meningitis	0.0	100	28
	Pneumonia	21.8	78.2	147
	Sepsis	2.1	97.9	48
Neonate	Birth asphyxia	2.4	97.6	800
	Low birth weight	25.1	74.9	231
	Other neonatal diseases	8.8	91.3	80
	Other respiratory diseases	5.6	94.4	36
	Sepsis of newborn	0.6	99.4	316

High Quality: GS1 (highest level of certainty), GS2A (high level of certainty), GS2B (high level of certainty)

Low Quality: GS3 (reasonable level of certainty), GS4 (unsupported)

^a^rows sum to 100%

### Quality of death certification

It was necessary to change UCOD for 45.5% of death certificates, with little difference by age group, sex, hospital, and cause of death (Table 4, Additional file 2). Child deaths due to gastroenteritis and colitis required more changes in the UCOD (60.0%) than all other causes for adults, children, and neonates. For most death certificates, changes in the UCOD were between 2 and 4 times more likely to have occurred due to a change in sequence than a change in diagnosis.

**Table 4:** Changes in the death certificate by top five causes of death

		Cause of death (%)				
Adults	Total (%)^a^	Stroke	Acute myocardial infarction	Other cardiovascular diseases	Other digestive diseases	Chronic obstructive pulmonary disease
Change necessary	45.5	52.3	45.5	44.3	46.8	41.8
Change in diagnosis	16.2	9.7	15.6	9.2	18.2	10.5
Change in sequence	29.4	42.5	30.0	35.1	28.6	31.4
Children	Total (%)^a^	Pneumonia	Sepsis	Gastroenteritis and colitis	Encephalitis	Meningitis
Change necessary	42.1	39.5	52.1	60.0	38.2	39.3
Change in diagnosis	13.6	12.2	10.4	22.9	0	14.3
Change in sequence	28.5	27.2	41.7	37.1	38.2	25

Change in sequence: study physician changed the sequence of events leading to death

Change in diagnosis: medical records led the study physician to change the UCOD

^a^All deaths

For the types of death certificate errors, nearly all physicians (95.6%) did not indicate the time interval between onset and death, 31.6% required a change in sequence, 13.9% required a change in diagnosis (Table 5). 99.1% of death certificates had any error, but 87.8% had any error other than the interval between onset and death not shown. The most frequent abbreviation for adults was “MI” (myocardial infarction) (9.8%), for children was “BR.” (breathing) (13.7%), and for neonates was “PNA” (perinatal asphyxia) (39.4%). No death certificates indicated neoplasm as a COD, and injuries were omitted from the error analysis because nearly all injuries did not indicate an intention.

**Table 5:** Frequency of types of death certificate errors

Type of Error	Error (%)
Multiple causes per line	41.5
Change in sequence necessary	31.6
Change in diagnosis necessary	13.9
Interval between onset and death not shown	95.6
Abbreviations used in certifying death	50.7
Leaving blanks between lines in part 1	7.2
Ill-defined condition entered as Underlying COD	33.2
Poisoning type or intent not indicated (*n* = 147)	99.3
Any error	99.1
Any error except interval	87.8

### Leading causes of in-hospital death

For adults, the top three causes of death were stroke (15.8%), acute myocardial infarction (14.4%), and other cardiovascular diseases (5.9%) (Table 6). Adult deaths also had a relatively large proportion of deaths that were “impossible to specify” (3.1%). For children the top three causes of death were pneumonia (31.7%), sepsis (10.4%), and gastroenteritis and colitis (7.6%) (Table 7). For neonates, the top three causes of death were birth asphyxia (52.8%), sepsis (20.9%), and low birth weight (15.2%) (Table 8).

**Table 6:** Top 20 underlying causes of death (assigned by study physician) for adults

Cause	Death (%)
Stroke	15.8
Acute myocardial infarction	14.4
Other cardiovascular diseases	5.9
Other digestive diseases	5.2
Chronic obstructive pulmonary disease	5.2
Cancers	4.7
Other infectious diseases	4.1
Other external causes of death	3.4
Impossible to specify	3.1
Nutritional diseases	3.0
Heart failure	2.9
Genitourinary diseases	2.7
Encephalitis	2.5
Ill-defined cause of death	2.3
Intentional poisoning	2.3
Other respiratory diseases	2.3
Other maternal	2.1
Paralytic ileus	2.1
Ischemic heart disease	1.9
Injuries and poisonings	1.8
Other (outside top 20)	12.2

**Table 7:** Top 10 causes of death (assigned by study physician) for children

Cause	Death (%)
Pneumonia	31.7
Sepsis	10.4
Gastroenteritis and colitis	7.6
Encephalitis	7.3
Meningitis	6.0
External causes of death	5.0
Protein-energy malnutrition	4.3
Other respiratory diseases	3.5
Other infectious diseases	2.6
Injuries and poisonings	2.4
Other (outside top 10)	19.1

**Table 8:** Top 5 causes of death (assigned by study physician) for neonates

Cause	Death (%)
Birth asphyxia	52.8
Sepsis of newborn	20.9
Low birth weight	15.2
Other neonatal diseases	5.3
Other respiratory diseases	2.4
Other (outside top 5)	3.4

## Discussion

Hospital physicians struggled to correctly complete the international MCCD form. Nearly all death certificates had some sort of error, even when the criteria were relaxed. These errors not only make it difficult for coders to record the information, but render the information meaningless for public health programs. Medical records were also of poor quality, with nearly half of diagnoses not supported by adequate clinical criteria.

While limited data exists for causes of hospital deaths in Bangladesh, the leading causes of in-hospital death in this study follow those from similar studies [15, 16]. However, the large proportion of adult deaths that were impossible to specify and child deaths due to sepsis was anomalous. Deaths that were impossible to specify can be attributed to physicians having either no or insufficient information to make a diagnosis. Similarly, physicians often diagnose sepsis when clinical features indicate illness of infectious origin, but they cannot make any definitive diagnosis either due to a lack of clear symptoms/signs or a lack of pathological support for making a specific diagnosis.

Not all errors in completing death certificates are detrimental to the value of the death certificate, but they can still compromise the accuracy of public health surveillance. For example, nearly all errors in assigning poisoning as a cause of death did not indicate whether the death was accidental or suicidal. While it can be inferred that the majority of deaths due to poisonings, such as organophosphate compound (OPC), are suicidal, certifiers need to indicate this intention on the death certificate because coders are unlikely to have culture-specific knowledge. Moreover, while the time interval between the onset of condition and death is not the most important part of the death certificate, it offers valuable insight into the likely sequence of causes leading to death in public health surveillance. Even if physicians are unable to determine a time interval, indicating “unknown” is an acceptable answer. We strongly encourage the certifying doctors to check all possible sources of information such as 1) double check with family members 2) check carefully all types of medical records (present and old) including prescriptions before putting “unknown” on the MCCD time interval. Many physicians use abbreviations in speech and clinical notes, but death certificates are legal documents and not always intended for a medical audience. It is important that physicians refrain from using abbreviations that can be confusing to coders, public health researchers, and families.

The format of the international form of the death certificate is more detailed and comprehensive than the present death certificates in Bangladesh, and many certifiers failed to adjust to the new form. Physicians often reported multiple causes of death as the underlying cause of death. It is unclear whether these errors were due to poor training, lack of understanding, or lack of attention. The previous death certificates also had no standard for the types of causes that could be written on the death certificate. Physicians struggled to adjust to removing ill-defined vocabulary such as “shock” and “cardiorespiratory failure.” Death certificates with such ambiguous wording can make it difficult to retrospectively assign a cause of death, especially when the quality of clinical records is poor.

Previous research has shown that education programs that train physicians in correctly filling death certificates can improve death certificate quality [7–12]. This study took place over 3 years with multiple opportunities for physicians to receive training. Given the numerous errors in the death certificates and poor clinical record keeping, it is unclear whether these training sessions were informative. Study physicians had a detailed knowledge of the international form of the death certificate as well as the culture of Bangladesh, so failure to properly communicate information about the death certificate may be due to poor instruction by study physicians or lack of attention or indifference by hospital physicians. It is also of immense importance that senior physicians guide and instruct junior physicians to strictly follow the MCCD. Study physicians noted that hospital physicians failed to adhere to the MCCD criteria until a departmental head emphasized the guidelines. It is possible that training would have been more effective for medical students and younger physicians who are new to the death certification process. A regular process of auditing may also result in higher quality death certificates.

Because this study comprised a large number of death certificates, we had to perform secondary analysis of errors automatically. We searched the text of transcribed death certificates for keywords to determine the frequency of ill-defined causes and abbreviations. While there was manual inspection to remove outliers, some death certificates likely evaded our analysis due to misspellings and other keywords. We were also unable to determine the frequency of death certificates with illegible handwriting because secondary analysis was conducted on death certificates in spreadsheet format. Lastly, the UCOD should be the last line on the death certificate, but because most physicians only used the first line, we only used this line to search for ill-defined UCOD.

## Conclusions

We found poor results when introducing the international form of the death certificate into Bangladesh. Potential reasons include poor training or lack of physician supervision. Physician certified death certificates implicitly serve as the gold standard in assigning cause of death, and they form the basis of mortality statistics that are used to inform policy as well as for research purposes [1, 17]. Urgent measures are needed to improve the quality of death certification in Bangladesh. The Bloomberg Data for Health Initiative is addressing this issue by introducing the International Form of the Medical Certificate of Cause of Death in four hospitals and conducting death certification training of master trainers and doctors.

## Additional files

Additional file 1: ICD-10 codes to text COD mapping. Three separate tables providing mapping from ICD-10 codes to text causes of death for adults, children, and neonates. (DOCX 19 kb)

Additional file 2: Changes in death certificate by age group, place of death, and sex. One table proving the percentage of death certificates that required a change in the UCOD due to a change in diagnosis or change in sequence by age group, place of death, and sex. (XLSX 9 kb)

## Abbreviations

COD: Cause of death
GS: Gold standard
ICD: International classification of diseases
icddr,b: International centre for diarrhoeal disease research, Bangladesh
MCCD: Medical certificate of cause of death
MDAF: Medical data and audit form
PHMRC: Population health metrics research consortium
UCOD: Underlying cause of death
VR: Vital registration
WHO: World Health Organization
