# Supplementary material for: The quality of medical death certification of cause of death in hospitals in rural Bangladesh: impact of introducing the International Form of Medical Certificate of Cause of Death
Source: BMC Health Serv Res. 2017 Oct 2;17:688. doi: 10.1186/s12913-017-2628-y (PMC5625830; doi:10.1186/s12913-017-2628-y)
Supplement: Supplementary file 1 — ICD-10 codes to text COD mapping. Three separate tables providing mapping from ICD-10 codes to text causes of death for adults, children, and neonates. (DOCX 19 kb) [file 12913_2017_2628_MOESM1_ESM.docx]

**ICD-10 Codes to Text COD Mapping**

| **Adult ICD-10 Code** | **Adult Text COD** |
| --- | --- |
| A09 | Gastroenteritis and colitis |
| A00-A08, A10-B99 | Other infectious diseases |
| C00-D48 | Cancers |
| D50-D89 | Blood diseases |
| E00-E90 | Nutritional diseases |
| F00-F99 | Mental and behavioral disorders |
| G04 | Encephalitis |
| G00-G03, G05-G99 | Other nervous system diseases |
| H00-H59 | Eye diseases |
| I21 | Acute myocardial infarction |
| I25 | Ischaemic heart disease |
| I50 | Heart failure |
| I61 | Intracerebral haemorrhage |
| I64 | Stroke |
| I00-I20, I22-I24, I26-I49, I51-I60, I62-I63, I65-I99 | Other cardiovascular diseases |
| J44 | Chronic obstructive pulmonary disease |
| J46 | Asthma |
| J00-J43, J45-J47, J48-J99 | Other respiratory diseases |
| K56 | Paralytic ileus |
| K00-K55, K57-K93 | Other digestive diseases |
| L00-L99 | Skin diseases |
| M00-M99 | Muscular diseases |
| N00-N99 | Genitourinary diseases |
| O72 | Postpartum haemorrhage |
| O00-O71, O73-O99 | Other maternal |
| P00-P96 | Neonatal diseases |
| Q00-Q99 | Chromosomal diseases |
| R99 | Impossible to specify |
| R00-R98 | Ill-defined cause of death |
| S00-T98 | Injuries and poisonings |
| V89 | Road traffic accident |
| X59 | Unknown injury |
| X68 | Intentional poisoning |
| V01-Y98 | Other external causes of death |

| **Child ICD-10 Code** | **Child Text COD** |
| --- | --- |
| A09 | Gastroenteritis and colitis |
| A41 | Sepsis |
| A00-A08, A10-A40, A43-B99 | Other infectious diseases |
| C00-D48 | Cancers |
| D50-D89 | Blood diseases |
| E43, E46 | Protein-energy malnutrition |
| E00-E42, E43-E45, E47-E90 | Other nutritional diseases |
| F00-F99 | Mental and behavioral disorders |
| G04 | Encephalitis |
| G03 | Meningitis |
| G00-G03, G06-G99 | Other nervous system diseases |
| H00-H59 | Eye diseases |
| I00-I99 | Cardiovascular diseases |
| J18 | Pneumonia |
| J00-J17, J19-J99 | Other respiratory diseases |
| K56 | Paralytic ileus |
| K00-K55, K57-K93 | Other digestive diseases |
| L00-L99 | Skin diseases |
| M00-M99 | Muscular diseases |
| N00-N99 | Genitourinary diseases |
| O00-O99 | Maternal |
| P00-P96 | Neonatal diseases |
| Q24 | Congenital malformations of heart |
| Q00-Q23, Q25-Q99 | Other chromosomal diseases |
| R99 | Impossible to specify |
| R00-R98 | Ill-defined cause of death |
| S00-T98 | Injuries and poisonings |
| V01-Y98 | External causes of death |

| **Neonate ICD-10 Code** | **Neonate Text COD** |
| --- | --- |
| A00-B99 | Infectious diseases |
| C00-D48 | Cancers |
| D50-D89 | Blood diseases |
| E00-E90 | Nutritional diseases |
| F00-F99 | Mental and behavioral disorders |
| G00-G99 | Nervous system diseases |
| H00-H59 | Eye diseases |
| I00-I99 | Cardiovascular diseases |
| J00-J99 | Respiratory diseases |
| K00-K93 | Digestive diseases |
| L00-L99 | Skin diseases |
| M00-M99 | Muscular diseases |
| N00-N99 | Genitourinary diseases |
| O00-O99 | Maternal |
| P07 | Low birth weight |
| P21 | Birth asphyxia |
| P36 | Sepsis of newborn |
| P00-P06, P08-P35, P37-P96 | Other neonatal diseases |
| Q00-Q99 | Chromosomal diseases |
| R00-R99 | Ill-defined cause of death |
| S00-T98 | Injuries and poisonings |
| V01-Y98 | External causes of death |
